# Emergency traffic adaptive MAC protocol for wireless body area networks based on prioritization

**DOI:** 10.1371/journal.pone.0225518

**Published:** 2019-12-02

**Authors:** Farhan Masud, Abdul Hanan Abdullah, Gaddafi Abdul-Salaam

**Affiliations:** 1 School of Computing, Faculty of Engineering, Universiti Teknologi Malaysia (UTM), Johor, Malaysia; 2 Department of Statistics & Computer Science, Faculty of Life Sciences Business Management, University of Veterinary and Animal Sciences, Lahore, Pakistan; 3 Department of Computer Science, Kwame Nkrumah University of Science & Technology, Kumasi, Ashanti Region, Ghana; Iowa State University, UNITED STATES

## Abstract

This paper proposes an emergency Traffic Adaptive MAC (eTA-MAC) protocol for WBANs based on Prioritization. The main advantage of the protocol is to provide traffic ranking through a Traffic Class Prioritization-based slotted-Carrier Sense Multiple Access/Collision Avoidance (TCP-CSMA/CA) scheme. The emergency traffic is handled through Emergency Traffic Class Provisioning-based slotted-CSMA/CA (ETCP-CSMA/CA) scheme. The emergency-based traffic adaptivity is provided through Emergency-based Traffic Adaptive slotted-CSMA/CA (ETA-CSMA/CA) scheme. The TCP-CSMA/CA scheme assigns a distinct, minimized and prioritized backoff period range to each traffic class in every backoff during channel access in Contention Access Period (CAP). The ETCP-CSMA/CA scheme delivers the sporadic emergency traffic that occurs at a single or multiple BMSN(s) instantaneously, with minimum delay and packet loss. It does this while being aware of normal traffic in the CAP. Then, the ETA-CSMA/CA scheme creates a balance between throughput and energy in the sporadic emergency situation with energy preservation of normal traffic BMSNs. The proposed protocol is evaluated using NS-2 simulator. The results indicate that the proposed protocol is better than the existing Medium Access Control (MAC) protocols by 86% decrease in packet delivery delay, 61% increase in throughput, and a 76% decrease in energy consumption.

## 1. Introduction

Wireless Body Area Networks (WBANs) offer a paradigm shift towards proactive management and early diagnosis of various diseases. The vital-signs data, such as body temperature, Heartbeat Rate (HR), Respiratory Rate (RR), Blood Pressure (BP), Electrocardiogram (ECG), Electroencephalogram (EEG), Electromyography (EMG), and pH-level are collected and analyzed by the use of different types of Bio-Medical Sensor Nodes (BMSNs) placed on/in the human body, or on wearable clothing. The heterogeneous-natured BMSNs (hereafter called BMSNs) generate various kinds of data packets containing the vital-signs information. Therefore, traffic prioritization is necessary during channel access due to the heterogeneous nature of vital-signs information [1–4]. Some Medium Access Control (MAC) protocols provide traffic prioritization such as [5–15]. However, some existing traffic priority MAC protocols use the standard slotted-Carrier Sense Multiple Access/Collision Avoidance (CSMA/CA) during contention to access the channel. In the standard slotted-CSMA/CA, the backoff period range of the first backoff repeats in the backoff period ranges of the next backoffs and the same backoff period range is assigned to all types of BMSNs in each backoff. On the contrary, some of them customize slotted-CSMA/CA for prioritized channel access. However, even in the customized slotted-CSMA/CA, the backoff period range of high priority traffic class is repetitively used in the backoff period range of low priority traffic class in each backoff and this situation causes non-prioritized channel access. Also, the assigned backoff period range remains unchanged in the second, third, fourth, and fifth backoffs. Furthermore, when the values of data generated by the BMSNs cross normal readings, then an emergency occurs. An emergency traffic is sporadic and should be delivered instantaneously [16, 17] since many delays risk the patient’s life. Therefore, identifying the emergency traffic is crucial in WBANs; it requires the highest priority [18, 19]. But sometimes emergency occurs at multiple BMSNs simultaneously [19]. Moreover, it is still necessary to consider normal traffic in emergency situation [20]. The existing MAC protocols such as [1, 2, 4, 16–19, 21–29] have only partially addressed the problem.

In WBAN, the patient’s body is observed by heterogeneous-natured BMSNs and thus, the applications consist of heterogeneous traffic rates which become variable in an emergency situation. For example, the BMSNs that are used to observe heartbeat, temperature, and blood pressure have low-rate traffic in a normal situation, but generate high rate of traffic in an emergency. The applications with low-rate traffic require less energy consumption [30] whereas those with high-rate emergency traffic demand high throughput. Moreover, in an emergency situation, many data packets are expired before transmission. But the communication channel is engaged with the transmission of dead data packets which is an extra burden on the network. Consequently, the performance of network is decreased in terms of packet delivery delay, throughput and energy consumption. Also, it is observed during experimentation that the energy consumption is increased and throughput is decreased due to high-rate emergency traffic. Therefore, dynamic adjustment of traffic is necessary to create a balance between throughput and energy [30]. Many prior work such as [1, 21, 22, 28, 30, 31] have proposed emergency traffic adaptive MAC protocols in WBANs to increase the network lifetime in WBANs. Again, different MAC protocols have been proposed to decrease energy consumption such as [32–47]. The overall performance of the WBANs is affected by the heterogeneous nature of BMSNs, sporadic emergency traffic, variations in the traffic generation rates, and the energy constraint.

In this paper, an Emergency Traffic Adaptive MAC (eTA-MAC) protocol is proposed for WBANs based on Prioritization in order to address the above mentioned gaps. The protocol is divided into three parts: Traffic Class Prioritization-based slotted-CSMA/CA (TCP-CSMA/CA) scheme, Emergency Traffic Class Provisioning-based slotted-CSMA/CA (ETCP-CSMA/CA) scheme, and Emergency-based Traffic Adaptive slotted-CSMA/CA (ETA-CSMA/CA) scheme.

The rest of this paper is organized as follows. Section 1 discusses related work. Section 2 presents design of the eTA-MAC protocol in detail. Section 3 presents performance evaluation of the proposed schemes. Finally, Section 4 concludes the paper.

## 2. Related work

Different MAC schemes have been proposed for emergency-based traffic adaptivity. These include the following:

In [2], Yoon et al. provide a traffic prioritization for diverse traffic types with preemptive channel allocation and non-preemptive data transmission in the allocated channels. However, the backoff period range of high priority class is repetitively used in the backoff period range of lower traffic class in each backoff which can also cause non-prioritized channel access. In addition, if a sporadic emergency alarm occurs during the occupation of Emergency Traffic Slot (ETS) by normal traffic, then the high priority is given to normal traffic [29]. Moreover, the number of ETS slots are not defined [3]. In [30], Rahman et al. propose A Traffic Load Aware Sensor (ATLAS) MAC to provide dynamic traffic adjustment, to preserve the energy of sensor nodes with low-rate traffic, and to create a balance between energy and throughput in case of high-rate traffic based on traffic load estimation. However, ATLAS does not consider the prioritized channel access for heterogeneous-natured BMSNs during contention. It is to be noted that different modes of operation in superframe based on traffic load results in a computational load [3]. Hence, non-prioritized traffic increases the collision and this collision may become worse in case of emergency situation. Therefore, all sensor nodes consume high energy and show very low throughput.

In [1], Anjum et al. present traffic Priority and Load Adaptive MAC (PLA-MAC) to provide contention-based traffic prioritization with high throughput, low packet delivery delay and less energy consumption. However, PLA-MAC uses the variable T_i_ instead of BE in the backoff period range equation. Therefore, the specific backoff period range assigned to the BMSNs of each traffic class remains unchanged in every backoff. Consequently, it consumes more energy in high traffic load. Furthermore, the backoff period ranges for high priority traffic classes are repetitively used in the backoff period ranges for low priority traffic classes. This situation results in a high collision among high and low priority packets. As a consequence, the performance of the whole scheme is reduced in terms of delay, throughput, and energy consumption. Similarly, it has fixed Emergency Time Slots (ETS) for emergency traffic. Such allocation results in wastage of resources in normal situations. In PLA-MAC, the emergency traffic is identified by using traffic-class value and traffic-generation rate which is not the practical approach. This is because the patient’s survival is based on HR, RR, temperature and BP vital-signs and thus, are considered by the doctors in an emergency situation [48–50]. In [21], Rezvani et al. aim to achieve the traffic prioritization with the adjustment of dynamic variations in heterogeneous traffic rates based on channel conditions to fulfil the requirements of BMSNs. However, in an emergency situation, the non-prioritized channel access during contention results in high collision and high energy consumption. Hence, the BMSNs with emergency or normal traffic drop the patient’s data while accessing the channel during contention [15].

In [3], Pandit et al. introduced an energy-efficient Multi-Constrained MAC (eMC-MAC) that provides contention-based traffic prioritization with emergency traffic handling in order to improve energy efficiency. However, the particular backoff period range assigned to the BMSNs of each traffic class remains the same in every backoff. It increases the packet loss ratio which affects the performance of the scheme in terms of packet delivery delay, on-time success ratio and energy consumption. Moreover, the repetitive usage of backoff period ranges of high priority classes in the backoff period ranges of low priority classes also increases the collision. The critical and reliability packets get the channel before emergency packets because they use zero as backoff number and emergency packets choose random backoff number from the backoff period range, i.e., [0–3]. Besides, no mechanism is provided to handle emergency at more than one BMSNs. In [18], Bhandari et al. provide a Priority-based Adaptive MAC (PA-MAC) to reduce the contention complexity during channel access with low energy consumption. PA-MAC tries to minimize the contention complexity but does not provide any mechanism from the start when there is only one phase in the Contention Access Period (CAP), and all traffic types contend to access the channel at the same level. This results in collisions of packets of different traffic classes causing delays in the overall traffic. Moreover, this delay becomes a reason to create the different number of sub-phases in the CAP. The delay further rises because more than one traffic types contend to access the channel in the second, third and fourth sub-phases which means contention complexity is still present in the second, third and fourth sub-phases.

In [29], Yu et al. propose a Contention over Reservation MAC (CoR-MAC) to provide contention over reservation mechanism with sporadic emergency traffic handling to maximize the channel utilization and minimize the delay. In CoR-MAC, the reserved slots for urgent data packets can be used by the sensor nodes with non-urgent traffic to transmit data which significantly improves channel utilization. However, collisions may increase when a sensor node with u_i_ traffic sporadically requires data transmission through its reserved slot S_i_ which is currently used by u_j_ due to the absence of u_i_ traffic. Hence, the dual reservation scheme improves the throughput, but it is not capable of removing the slot allocation conflict between multiple sensor nodes with urgent traffic. In [4], Rasheed et al. introduce a Priority Guaranteed MAC (PG-MAC) to provide contention-based prioritized channel access with low packet delivery delay and less energy consumption. But it uses D_type_ instead of BE in the backoff period range equation. Therefore, the backoff period range that is assigned to the BMSN of any traffic class remains the same in all backoffs. Furthermore, the backoff period range of the high priority class is repetitively used in the backoff period ranges of the low priority classes. In addition, the consideration of emergency traffic on the basis of traffic class value and traffic generation rate is not the real-time practical approach The reason is that the BMSNs with lowest traffic class value and highest traffic generation rate are not considered by doctors in an emergency situation. Hence, the performance of PG-MAC decreases in terms of throughput and energy consumption.

## 3. Proposed eTA-MAC protocol

### 3.1. Overview

The proposed eTA-MAC focuses on the contention phase of beacon-enabled mode of IEEE 802.15.4 standard. In the proposed eTA-MAC protocol, TCP-CSMA/CA, ETCP-CSMA/CA and ETA-CSMA/CA schemes collaborate with each other to increase throughput and decrease delay and energy consumption. Data packets are classified into five different classes, namely, Emergency Data Packet (EDP) (readings of HR, RR, Temperature and BP vital-signs which cross the normal readings), Critical Data Packet (CDP) (cannot tolerate much losses and need to be delivered within specific time-frame e.g., EEG and ECG), Reliability-constrained Data Packet (RDP) (to be delivered with minimum losses but not within specific time-frame e.g., HR and RR), Delay-constrained Data Packet (DDP) (can tolerate some losses but need to be delivered within specific time-frame e.g., telemedicine video imaging), and Normal Data Packet (NDP) (can tolerate losses and do not have any time-constraint e.g., BP and temperature).

**Fig 1:**
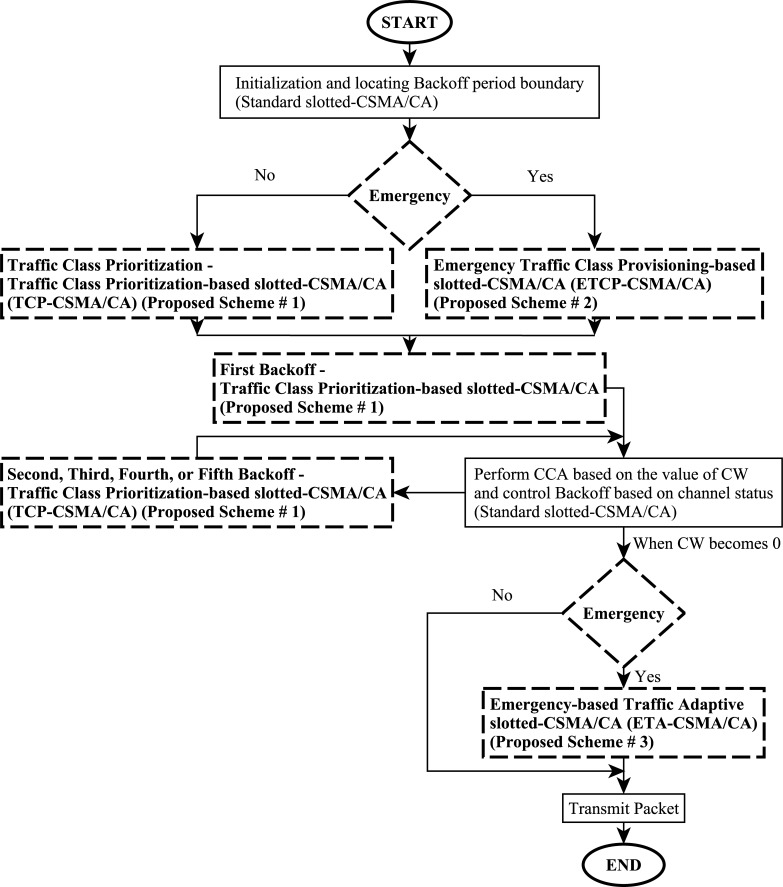
Framework of emergency Traffic Adaptive MAC (eTA-MAC) protocol for WBANs based on prioritization, the dotted blocks show the research contributions.

The framework of eTA-MAC protocol is shown in (Fig 1). Initially, all BMSNs perform Traffic Class Prioritization after locating the backoff period boundary. Following this, each BMSN performs the First Backoff of TCP-CSMA/CA and then writes in full (CCA) based on the value of CW and may perform Second, Third, Fourth, or Fifth Backoff of TCP-CSMA/CA before packet transmission. Additionally, in an emergency situation, all the BMSNs perform ETCP-CSMA/CA scheme after locating backoff period boundary. Then, each BMSN performs the First Backoff of TCP-CSMA/CA. After this, the BMSN performs CCA based on the value of CW and may perform Second, Third, Fourth, or Fifth Backoff of TCP-CSMA/CA before packet transmission. In an emergency situation, when the value of CW becomes 0, all the BMSNs perform Emergency based Traffic Adaptive slotted-CSMA/CA before packet transmission.

### 3.2 Design of TCP-CSMA/CA scheme

In the TCP-CSMA/CA scheme, a distinct, minimized and prioritized backoff period range is assigned to each Traffic Class (TC). The backoff period range (i.e., assigned to a particular BMSN) is not repeated in any backoff. Therefore, each BMSN uses a distinct backoff period range. Only the first backoff period range in the first backoff starts from zero; the rest of do not. The following backoff period ranges are proposed to achieve the above-mentioned targets.

Backoff Period Range used in first backoff:

$${[T}_{CV}\;2^{\left(BE+1\right)}To\;2^{BE}+4T_{CV}+1]$$ (1)

Backoff Period Range used in second backoff:

$$\left[2^{BE}(T_{CV}+1)To\;2^{BE}+4T_{CV}+3\right]$$ (2)

Backoff Period Range used in third backoff:

$$\left[2^{BE}(T_{CV}+1)-4T_{CV}\;To\;2^{BE}+4T_{CV}+3\right]$$ (3)

Backoff Period Range used in fourth backoff:

$$\left[2^{\left(BE-1\right)}+4(T_{CV}+1)To\;2^{BE}+4T_{CV}-1\right]$$ (4)

Backoff Period Range used in fifth backoff:

$$\left[2^{\left(BE-1\right)}+4T_{CV}\;To\;2^{\left(BE-1\right)}+4T_{CV}+3\right]$$ (5)

where BE is backoff exponent and T_CV_ represents traffic class value. The proposed scheme introduces four traffic classes based on WBANs traffic classification that are Critical Traffic Class (CTC) for BMSNs with CDPs, Reliability Traffic Class (RTC) for BMSNs with RDPs, Delay Traffic Class (DTC) for BMSNs with DDPs and Non-constrained Traffic Class (NTC) for BMSNs with NDPs. The highest priority assigns to the CTC, the second highest priority assigns to RTC, the third highest priority assigns to DTC, and the lowest priority is assigned to NTC.

The contention is distributed among five backoffs. Initially, TCP-CSMA/CA scheme initializes the variables NB to 0 and CW to 2. It also uses constants; macMinBE and aMaxBE to represent the minimum and the maximum number of backoffs respectively. The value of macMinBE is 1 and that of aMaxBE is 5. Then, BMSN verifies that the value of Battery Life Extension (BLE) (i.e., used to determine the duration of CAP which is equivalent to six complete backoff periods, if BLE = true) is either true or false. In TCP-CSMA/CA scheme, BLE is initialized to false. Hence, in the first backoff, the value of BE is 1. Afterwards, the MAC sublayer of BMSN locates the next backoff period boundary. It further verifies whether BMSN is with CDP or not. If it is, then, 0 is assigned to T_CV_. Otherwise, it verifies whether it is with RDP. If yes, then, 1 is assigned to T_CV_. However, if it is with DDP, then, 2 is assigned to T_CV_, else 3 is assigned to T_CV_. Later on, the BMSN waits for a random number of backup periods which is selected from the backoff period range computed by using Eq 1 in the first backoff.

It is worth emphasizing that every BMSN performs two times CCA in each backoff while trying to access the channel. Therefore, the MAC sublayer of BMSN requests its PHY sublayer of BMSN to perform CCA at the backoff period boundary to ensure collision-free channel access. If the channel is idle, then, the value of CW is decreased by 1. Afterwards, the BMSN verifies whether the value of CW is 0. If yes, then, the MAC sublayer of BMSN again requests its PHY sublayer to perform CCA again. But if the value of CW becomes 0, then, BMSN gets the channel and transmits the patient’s data.

In case BMSN finds a busy channel, then its MAC sublayer resets CW to 2 and increases the values of NB and BE by 1. But the value of BE should not exceed the contention threshold value of aMaxBE. Hence, it verifies whether the value of NB is greater than the value of macMaxCSMABackoffs or not. In case the value is higher, then BMSN drops the packet, and the TCP-CSMA/CA is terminated with the status of channel access failure. If the value of NB is less than or equal to the value of macMaxCSMABackoffs, then the BMSN goes for second backoff.

Similarly, in the second backoff, each BMSN selects the random number from the backoff period range computed by Eq 2. Hence, in second backoff, each traffic class uses a distinct and prioritized backoff period range to select the random backoff number. If a BMSN fails to access the channel in the second backoff then it goes for the third backoff. In the third, fourth or fifth backoff, each BMSN chooses the random number from the backoff period range computed by using Eqs 3, 4 or 5 respectively. The TCP-CSMA/CA scheme assigns a distinct and prioritized backoff period range to each traffic class during contention for channel access in each backoff. In standard slotted-CSMA/CA scheme, same backoff period range is assigned to all BMSNs during contention for channel access which increases the collision rate and the delay, decreases the throughput, and high energy is consumed by BMSNs. Thus, the proposed TCP-CSMA/CA scheme, decreases the packet collision rate, packet delivery delay, and energy consumption, and increases the throughput. It also provides the prioritized channel access to BMSNs in the CAP. Furthermore, Tables 1 and 2 show the backoff period ranges calculation based on the propose Equations in normal and emergency situations.

**Table 1:** Traffic class-wise computed backoff period ranges used by BMSNs for the selection of random backoff number in each backoff during a normal situation.

T_CV_	1^st^ Backoff, BE = 1			2^nd^ Backoff, BE = 2			3^rd^ Backoff, BE = 3			4^th^ Backoff, BE = 4			5^th^ Backoff, BE = 5		
	Eq 1		TC-wise BPRs	Eq 2		TC-wise BPRs	Eq 3		TC-wise BPRs	Eq 4		TC-wise BPRs	Eq 5		TC-wise BPRs
	T_CV_ 2^(BE+1)^	2^BE^ +4T_CV_ + 1		2^BE^ (T_CV_ + 1)	2^BE^ + 4T_CV_ + 3		2^BE^ (T_CV_ + 1) – 4T_CV_	2^BE^ + 4T_CV_ + 3		2^(BE-1)^ + 4(T_CV_ + 1)	2^BE^ + 4T_CV_− 1		2^(BE-1)^ + 4T_CV_	2^(BE-1)^ + 4T_CV_ + 3	
0 (BMSNs with CDPs)	0	3	[0–3]	4	7	[4–7]	8	11	[8–11]	12	15	[12 – 15]	16	19	[16–19]
1 (BMSNs with RDPs)	4	7	[4–7]	8	11	[8–11]	12	15	[12–15]	16	19	[16 – 19]	20	23	[20–23]
2 (BMSNs with DDPs)	8	11	[8–11]	12	15	[12–15]	16	19	[16–19]	20	23	[20 – 23]	24	27	[24–27]
3 (BMSNs with NDPs)	12	15	[12–15]	16	19	[16–19]	20	23	[20–23]	24	27	[24 – 27]	28	31	[28–31]

BE = Backoff Exponent, T_CV_ = Traffic Class Value, BPR = Backoff Period Range, and Eq. = Equation

**Table 2:** Traffic class-wise computed backoff period ranges used by BMSNs for the selection of random backoff number in each backoff during an emergency situation.

T_CV_	1^st^ Backoff, BE = 1			2^nd^ Backoff, BE = 2			3^rd^ Backoff, BE = 3			4^th^ Backoff, BE = 4			5^th^ Backoff, BE = 5		
	Eq 1		TC-wise BPRs	Eq 2		TC-wise BPRs	Eq 3		TC-wise BPRs	Eq 4		TC-wise BPRs	Eq 5		TC-wise BPRs
	T_CV_ 2^(BE+1)^	2^BE^ + 4T_CV_ + 1		2^BE^ (T_CV_ + 1)	2^BE^ + 4T_CV_ + 3		2^BE^ (T_CV_ + 1)– 4T_CV_	2^BE^ + 4T_CV_ + 3		2^(BE-1)^ + 4(T_CV_ + 1)	2^BE^ + 4T_CV_− 1		2^(BE-1)^ + 4T_CV_	2^(BE-1)^ + 4T_CV_ + 3	
0 (BMSNs with EDPs)	0	3	[0–3]	4	7	[4–7]	8	11	[8–11]	12	15	[12–15]	16	19	[16–19]
1 (BMSNs with CDPs)	4	7	[4–7]	8	11	[8–11]	12	15	[12–15]	16	19	[16–19]	20	23	[20–23]
2 (BMSNs with RDPs)	8	11	[8–11]	12	15	[12–15]	16	19	[16–19]	20	23	[20–23]	24	27	[24–27]
3 (BMSNs with DDPs)	12	15	[12–15]	16	19	[16–19]	20	23	[20–23]	24	27	[24–27]	28	31	[28–31]
4 (BMSNs with NDPs)	16	19	[16–19]	20	23	[20–23]	24	27	[24–27]	28	31	[28–31]	32	35	[32–35]

BE = Backoff Exponent, T_CV_ = Traffic Class Value, BPR = Backoff Period Range, and Eq. = Equation

### 3.3 Design of ETCP-CSMA/CA scheme

The ETCP-CSMA/CA scheme is designed to deliver instantaneously, sporadic emergency traffic that occurs at the BMSNs, with minimum delay without ignoring normal traffic in CAP. The patient’s survival is based on HR, RR, Temperature and BP vital-signs and thus, are considered by doctors in an emergency situation [48–50]. The HR and RR are monitored by BMSNs with RDPs. The BMSNs with NDPs monitor Temperature and BP. In an emergency situation, the BMSNs that monitor HR, RR, Temperature and BP vital-signs of a patient’s body are considered as Expected Emergency BMSNs (EE-BMSNs). The EE-BMSNs are BMSN_HR (i.e., Pulse Oximeter used to monitor the HR), BMSN_RR (i.e., Respiratory Rate Monitor), BMSN_TM (i.e., Thermometer used to monitor temperature) and BMSN_BP (i.e., Blood Pressure Monitor). This study also introduces EDPs that are generated by EE-BMSNs in case of emergency. This study introduces an Emergency Traffic Class (ETC) that is generated dynamically, the highest priority is assigned to ETC, and the priority of all TCs go down one level dynamically in an emergency situation. Therefore, in an emergency situation, the highest priority assigns to ETC, the second highest priority assigns to CTC, the third highest priority assigns to RTC, the fourth highest priority is assigns to DTC, and the lowest priority is assigns to NTC. Furthermore, the ETC is removed dynamically when an emergency resolves and the priority of all TCs go back to normal (illustrated in Section 2.2).

Each BMSN declares four emergency flags at its MAC layer to handle emergency traffic. These emergency flags are used to handle emergency traffic at BMSN_HR, BMSN_RR, BMSN_TM and BMSN_BP, respectively. Initially, each BMSN initializes all of its emergency flags to false. The proposed ETCP-CSMA/CA scheme handles an emergency at BMSN level without involving BC. If an emergency occurs at any EE-BMSN, then the EE-BMSN broadcasts EmergencyAlert beacon which is received by all the BMSNs of the network. So, all the BMSNs of the network set their flags to true, based on the specific EE-BMSN which broadcasts the EmergencyAlert beacon. After flag settings, ETCP-CSMA/CA is used to assign the prioritized T_CV_ to all the BMSNs based on traffic class prioritization in an emergency situation. ETCP-CSMA/CA then integrates with the proposed TCP-CSMA/CA scheme (illustrated in Section 2.2). Therefore, the BMSNs perform the initial steps of TCP-CSMA/CA scheme up to locating the next backoff period boundary. Afterwards, it is to verify whether emergency occurs at single or multiple EE-BMSNs.

If emergency occurs at a single BMSN, then each one of them checks the emergency flag that is true during contention. The lowest T_CV_ is assigned to BMSN_HR, BMSN_RR, BMSN_TM or BMSN_BP based on the verification of their respective flags. The priorities of the remaining BMSNs are then shifted one level down. However, the notion behind this is to create ETC dynamically, to change the traffic class of any single EE-BMSN and to assign the lowest T_CV_ to that particular EE-BMSN. For example, in a normal situation, BMSN_HR is the member of RTC, but in an emergency situation, BMSN_HR leaves the membership of RTC and becomes a member of ETC dynamically. Therefore, BMSN_HR gets the lowest T_CV_ (i.e., zero). However, when an emergency situation completes, the BMSN_HR leaves the group of ETC and again becomes a member of RTC dynamically.

If an emergency occurs at any two EE-BMSNs at the same time while contending to access the channel in the CAP, each BMSN verifies which of the two emergency flags are true during contention. Based upon this verification, the highest priority is given to those two EE-BMSNs that have the emergency, and the priorities of the remaining BMSNs are shifted one level down during contention. For example, in a normal situation, BMSN_HR and BMSN_RR are the members of RTC. But both BMSN_HR and BMSN_RR leave the membership of RTC and become the member of ETC dynamically if emergency occurs in both of them. Therefore, both BMSN_HR and BMSN_RR get lowest T_CV_ (i.e., zero). After the emergency is resolved at both BMSN_HR and BMSN_RR, they leave the membership of ETC and again become members of RTC dynamically.

If an emergency occurs at any three EE-BMSNs at the same time during contention for channel access in a CAP, then each BMSN verifies the values of any three emergency flags that are true. Based upon this verification, the highest priority is assigned to those three EE-BMSNs., and the priorities of the remaining BMSNs are shifted one level down. However, the primary objective of ETCP-CSMA/CA is to create an ETC dynamically, and change the TC of any three EE-BMSNs in an emergency situation. For example, in a normal situation, BMSN_HR and BMSN_RR are the members of RTC, and BMSN_TM is a member of NTC. But when emergency occurs at BMSN_HR, BMSN_RR and BMSN_TM, then all of them leave the membership of their corresponding traffic classes and become members of ETC dynamically. Therefore, BMSN_HR, BMSN_RR and BMSN_TM get the lowest T_CV_ (i.e., zero) during an emergency situation. When the emergency is completed at BMSN_HR, BMSN_RR and BMSN_TM, then they leave the membership of ETC. The BMSN_HR and BMSN_RR return to be members of RTC while BMSN_TM becomes a member of NTC dynamically.

Similarly, if an emergency occurs at all EE-BMSNs then the highest priority is assigned to EE-BMSNs. The highest priority is assigned to EE-BMSNs by verifying which EE-BMSN with EDP contends to access the channel. Therefore, if BMSN_HR, BMSN_RR, BMSN_TM or BMSN_BP is with EDP, then 0 is assigned to its T_CV_. For instance, if BMSN_HR with EDP contends to access the channel, then 0 is assigned to its T_CV_ which is further used in TCP-CSMA/CA scheme to assign the lowest backoff period range to BMSN_HR. BMSN_HR gets the lowest backoff number based on lowest backoff period range, and as a result BMSN_HR achieves the highest priority to access the channel. In addition, the proposed ETCP-CSMA/CA scheme also verifies other types of BMSNs with different types of packets. Therefore, it is to check either BMSN is with CDP or not. In case BMSN is with CDP then 1 is assigned to its T_CV_. If it is with RDP, then 2 is assigned to its T_CV_. If it is with DDP, then 3 is assigned to its T_CV_. Or if BMSN is with NDP, then 4 is assigned to its T_CV_. Afterwards, goes to the TCP-CSMA/CA scheme (described in Section 2.2).

Therefore, a distinct backoff period range is assigned to ETC in every backoff. Moreover, the lowest and prioritized backoff period range is assigned to ETC in every backoff to grant the highest priority to ETC. Additionally, distinct, minimized and prioritized backoff period ranges are also assigned to the BMSNs with normal data packets in each backoff. Hence, there is a high probability of emergency traffic transmission before the transmission of all normal traffic types. The normal traffic types also get the transmission opportunity and an emergency situation is handled at the BMSNs level.

The emergency resolved situation is also handled at BMSN level without involving BC. It is assumed that all BMSNs of the network receive the EmergencyResolve beacon, whenever it is broadcast by any EE-BMSN in the network. Then, all the BMSNs of the network set their flags to false based on the specific EE-BMSN which broadcasts the EmergencyResolve beacon. After flag settings, the ETCP-CSMA/CA assigns the prioritized T_CV_ to all the BMSNs, based on traffic class prioritization and moves to the proposed TCP-CSMA/CA scheme (presented in Section 2.2). Therefore, the BMSNs perform the initial steps of TCP-CSMA/CA scheme up to locating the next backoff period boundary. Then, it verifies whether emergency resolves at single, or at multiple EE-BMSNs. If emergency resolves at a single EE-BMSN but present at three EE-BMSNs, then it repeats the process as explained earlier in this Section for emergency occurrence at three EE-BMSNs. Similarly, if emergency is resolved at any two EE-BMSNs but present at two EE-BMSNs, then it repeats the process as explained earlier in this Section for emergency occurrence at any two EE-BMSNs. Finally, if emergency is resolved at any three EE-BMSNs but persists at a single EE-BMSN, then it repeats the process as explained earlier in this Section for emergency occurrence at any single EE-BMSNs. In case emergency is resolved from all the EE-BMSNs then the value 0, 1, 2, or 3 is assigned to T_CV_ of the particular BMSN based on its type of data packet. Finally, it moves to the TCP-CSMA/CA scheme.

### 3.4 Design of ETA-CSMA/CA scheme

The proposed ETA-CSMA/CA scheme provides lifetime management system for all types of data packet in an emergency situation. In the TCP-CSMA/CA scheme, when CW becomes zero, then the BMSN executes the ETA-CSMA/CA scheme before packet transmission.

In the ETA-CSMA/CA scheme, a variable named Pkt_NLT_ (Packet Normal LifeTime) is declared in all BMSNs to manage the lifetime of each normal data packet. A variable named Pkt_ELT_ (Packet Emergency LifeTime) is defined in all EE-BMSNs to manage the lifetime of each emergency data packet. Then, a variable MHR_timeStamp_ is defined in the protocol specific packet header (i.e., hdr_lrwpan). The scheme uses LR-WPANs (Low Rate-Wireless Personal Area Network) protocol. The MHR_timeStamp_ is used to store the data packet’s generation time, based on the virtual simulation time. A variable named Pkt_CLT_ (Packet Current LifeTime) is used to store the time taken by the data packet from generation to transmission. A pointer wph is used to access the protocol specific packet header (i.e., hdr_lrwpan) of the particular data packet.

Each BMSN initializes its variable Pkt_NLT_ to a specific time in seconds (i.e., the normal lifetime based on its application). Also, each EE-BMSN initializes its variable Pkt_ELT_ to a particular time in seconds (i.e., the emergency lifetime based on its application). Afterwards, a CURRENT_TIME (i.e., current virtual simulation time) is assigned to a variable MHR_timeStamp_ during the construction of each normal or emergency data packet's MAC Protocol Data Unit (MPDU) (i.e., MAC frame). Later on, the starting address of the current packet's protocol-specific header (i.e., HDR_LRWPAN(packet)) is assigned to pointer wph. HDR_ LRWPAN is a function used to access the starting address of input packet’s protocol-specific header. Then, it is to confirm that the current packet does not belong to BC and it is not a command packet. If the current packet is the data packet from BC or a command packet from any BMSN or BC, then this packet is transmitted. Therefore, the packet’s current lifetime is calculated by using Eq 6 as follows:

$${Pkt}_{CLT}=CURRENT\_TIME+mac->txtime(packet)–wph->{MHR}_{timeStamp}$$ (6)

where the statement mac->txtime(packet) returns the time that is taken by the sender BMSN to transmit all bits of the current packet plus the time required during link propagation delay (i.e., the time taken by bits to travel from sender to receiver). The link propagation delay is ignorable because for wireless communications the propagation speed is equivalent to the speed of light.

If the current data packet is from BMSN_HR, the emergency flag for HR is on and the packet’s current lifetime is more than the predefined emergency lifetime, then, this dead emergency data packet is dropped. Else, it verifies that the current data packet is from BMSN_RR, the emergency flag for RR is on, and the packet’s current lifetime is more than the predefined emergency lifetime. If it is true, then the dead emergency data packet is dropped. However, if the current data packet is from BMSN_TM, and its current calculated lifetime is greater than the predefined emergency lifetime in case of an emergency at BMSN_TM, then, the dead emergency data packet is dropped. Otherwise, if the current data packet is from BMSN_BP, the emergency flag for BP is on (i.e., BMSN_BP has emergency data packets), and the packet’s current calculated lifetime is more than the predefined emergency lifetime, then, the dead emergency data packet is dropped. If the current data packet is from one of the EE-BMSNs in an emergency situation and if is still alive or valid for transmission, then, this emergency data packet is transmitted. Thus, the transmission of the emergency data packets is controlled in terms of lifetime.

Finally, if the current data packet is from any BMSN with normal traffic and the packet’s current lifetime crosses the predefined normal lifetime in an emergency situation, then this normal data packet is dropped. Otherwise, it is transmitted. Hence, the normal data packets are dropped by the BMSNs which are dead before transmission in an emergency situation. The scheme also provides a mechanism to handle the transmission of normal data packets in terms of lifetime.

An algorithm for ETA-CSMA/CA scheme is used to provide the lifetime management system for all types of data packets in an emergency situation. Therefore, in case of emergency, when the lifetime of emergency data packets is decreased, and emergency traffic rate is increased, the ETA-CSMA/CA scheme stops the transmission of dead data packets (whose lifetime is expired) either from EE-BMSNs or BMSNs with normal traffic. Hence, the dynamic variations in heterogeneous traffic rates are adjusted dynamically, the energy of BMSNs with normal traffic is preserved, and a balance is created between throughput and energy. Also, the performance of ETA-CSMA/CA scheme is improved in terms of packet delivery delay, throughput and energy consumption. The algorithm for the ETA-CSMA/CA scheme is given as follows:

**Algorithm 1: ETA-CSMA/CA**: Emergency-based Traffic Adaptive slotted-CSMA/CA

**Notations**

Pkt_NLT_: Packet Normal LifeTime

Pkt_ELT_: Packet Emergency LifeTime

Pkt_CLT_: Packet Current LifeTime

MPDU: MAC Protocol Data Unit

wph: It is a pointer used to access the protocol specific packet header of the particular data packet

HDR_LRWPAN: A function used to access the starting address of the input packet’s protocol-specific header

mac->index_: mac is a pointer that returns the ID of the current BMSN

BC_ID: Unique ID of BC

MHR_timeStamp_: It stores the data packet’s generation time based on the virtual simulation time

mac->txtime(packet): It returns the time required by the sender BMSN to transmit all bits of the current packet plus the time required during link propagation delay

BMSN_HR_ID: Unique ID of BMSN_HR

BMSN_RR_ID: Unique ID of BMSN_RR

BMSN_TM_ID: Unique ID of BMSN_TM

BMSN_BP_ID: Unique ID of BMSN_BP

eFlag_HR: An emergency flag used to handle emergency traffic at BMSN_HR

eFlag_RR: An emergency flag used to handle emergency traffic at BMSN_RR

eFlag_TM: An emergency flag used to handle emergency traffic at BMSN_TM

eFlag_BP: An emergency flag used to handle emergency traffic at BMSN_BP

**Input:**

Pkt_NLT_, Pkt_ELT_, BMSN_i__ID, BMSN_i_, packet, eFlag_HR, eFlag_RR, eFlag_TM, eFlag_BP

**Process**

    1. **Set** Pkt_NLT_ ← Assign the application-specific value (i.e., time in seconds) during the instantiation of BMSN_i_

    2. **Set** Pkt_ELT_ ← Assign the application-specific value (i.e., time in seconds) during the instantiation of BMSN_i_

    3. **Set** MHR_timeStamp_ ← CURRENT_TIME

        //Assign the starting address of current packet's protocol specific header to pointer "wph" in the following statement

    4. **Set** wph ← HDR_LRWPAN (packet)

    5. **if** (mac->index_ ! = BC_ID) AND (wph->MHR_timeStamp_ ! = 0.0) **then**

    6.    Pkt_CLT_ = CURRENT_TIME + mac->txtime(packet)–

            wph->MHR_timeStamp_

    7.    **if** ((mac->index_ = = BMSN_HR_ID) AND (eFlagHR == true) AND (Pkt_CLT_ > Pkt_ELT_)) **then**

    8.          Drop the data packet

    9.    **else if** ((mac->index_ = = BMSN_RR_ID) AND (eFlagRR = = true) AND (Pkt_CLT_ > Pkt_ELT_)) **then**

    10.          Drop the data packet

    11.    **else if** ((mac->index_ = = BMSN_TM_ID) AND (eFlagTM = = true) AND (Pkt_CLT_ > Pkt_ELT_)) **then**

    12.          Drop the data packet

    13.    **else if** ((mac->index_ = = BMSN_BP_ID) AND (eFlagBP = = true) AND (Pkt_CLT_ > Pkt_ELT_)) **then**

    14.          Drop the data packet

    15.    **else if** ((mac->index_ = = BMSN_HR_ID) OR (mac->index_ = = BMSN_RR_ID) OR

                    (mac->index_ = = BMSN_TM_ID) OR (mac->index_ = = BMSN_BP_ID)) AND

                    ((eFlagHR = = true) OR (eFlagRR = = true) OR (eFlagTM = = true) OR (eFlagBP = = true)) **then**

    16.        Transmit the data packet

    17.    **else if** (mac->index_ = = BMSN_i__ID) AND ((eFlagHR = = true) OR (eFlagRR = = true) OR

                    (eFlagTM = = true) OR (eFlagBP = = true)) AND (Pkt_CLT_ > Pkt_NLT_) **then**

    18.          Drop data packet

    19.    **else**

    20.          Transmit the data packet

    21.    **end if**

    22. **end if**

**Output:** It drops the dead emergency and normal data packets before transmission in an emergency situation.

## 4 Performance evaluation

Extensive simulations are conducted in NS-2 to evaluate performance of the eTA-MAC protocol. The TCP-CSMA/CA and ETCP-CSMA/CA schemes of eTA-MAC are compared with PLA-MAC [1], eMC-MAC [3] and PG-MAC [4] while ETA-CSMA/CA scheme of eTA-MAC is compared with ATLAS [30] and PLA-MAC [1]. The emergency occurrence at different EE-BMSNs is compared. All the comparisons were done in terms of average packet delivery delay, throughput, and energy consumption. The results show that eTA-MAC performs better than the benchmark protocols.

### 4.1 Simulation model

In the proposed eTA-MAC protocol, fourteen BMSNs are deployed on the human body. These BMSNs are directly connected with the on-body local base station Body Coordinator (BC). All BMSNs transmit observed data packets to BC using contention to access the channel in the CAP. It assumes that BMSNs have limited processing power and energy supply. But BC has the highest processing power and has an external power supply. The simulation parameters used for eTA-MAC protocol are presented in Table 3.

**Table 3:** Simulation parameters.

Parameter	Value	Parameter	Value
Operating Carrier Frequency	2.4 GHz	Base Slot Duration	60 symbols
Channel Data Rate	250kbps	Sending Data Rate	62.5 kbps
A Slot Duration	15.36 msec	Beacon Interval Duration	491.52 msec
Superframe Duration	245.76 msec	Inactive Period Duration	245.76 msec
Number of Superframe Slots	16	MAC Data Payload	102 bytes
Beacon Order (BO)	5	Max PHY Packet Size	127 bytes
Superframe Order (SO)	4	TurnaroundTime	12 symbols
a CCA Time	8 symbols	UnitBackoffPeriod	20 symbols
Max Frame Retries	3	MacAckWaitDuration	55
Number of nodes	14	Body Coordinator	1
Minimum Backoff Exponent (BE)	1	Maximum BE	5
Battery Life Extension (BLE)	False	Synchronization Mode	Beacon-Enabled
Traffic Type	CBR	Initial Power	100 W
MaxCSMABackoffs	4	Power Consumed in Transmission state	0.027–0.22 W
Power Consumed in the Reception state	0.0018 W	Power Consumed during Transition	0.0004 W
Power consumed in a Sleep state	0.000005 W	Time Required for Transition	0.0008 sec
Simulation Time	2000 sec	Topology	Star

### 4.2 Simulation results

The performance of the proposed eTA-MAC protocol is presented with respect to different number of BMSNs in normal or emergency situation, the emergency occurrence at various EE-BMSNs conducted through varying time in seconds and different traffic loads which are from 1 packet/second to 7 packets/second. The BMSNs are varied from 1 to 14 in a normal situation while in an emergency situation, the number of BMSNs with emergency traffic is 4 and BMSNs with normal traffic are 10. Moreover, the probability of simultaneous emergency occurrence at multiple EE-BMSNs is 64%.

#### 4.2.1 Average packet delivery delay

**Fig 2:**
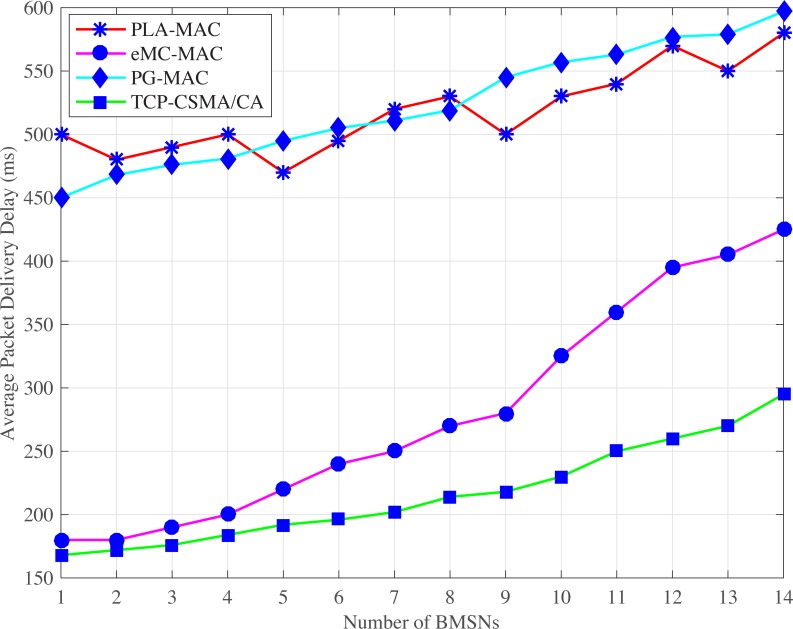
Average packet delivery delay versus number of BMSNs in normal situation.

**Fig 3:**
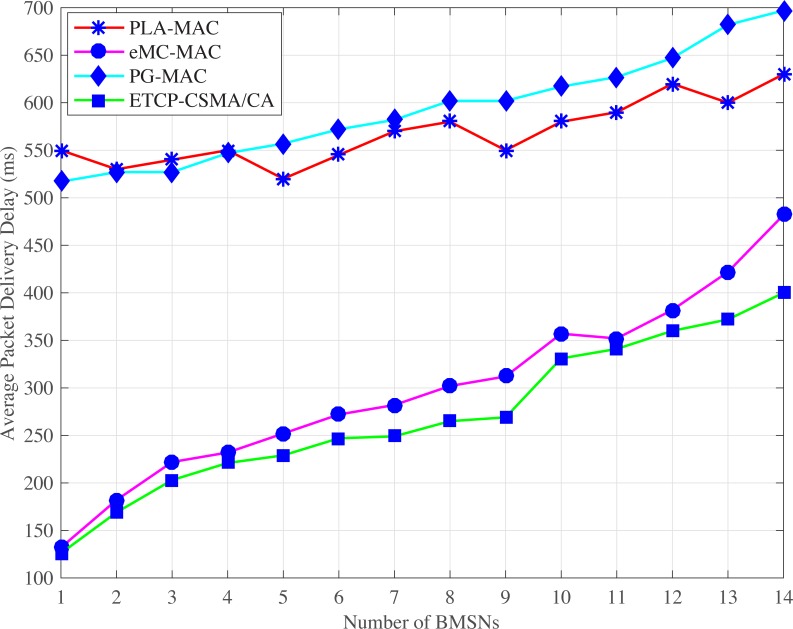
Average packet delivery delay versus number of BMSNs in emergency situation.

Figs 2 and 3 show the average packet delivery delay comparison of MAC protocols in normal and emergency situations. The PLA-MAC indicates very high average packet delivery delay. In PLA-MAC, the BMSNs with emergency packets get the lowest backoff period range during contention. Therefore, the BMSNs with emergency packets get the channel faster and more frequently. BMSNs with other types of data packets get the channel later and thereby, increase the delay for BMSNs with low priority data packets. In PLA-MAC, each BMSN uses the backoff period range which starts from zero. Thus, the low priority data packets can access the channel before the emergency data packets which also delays the emergency data packets. Furthermore, the assigned backoff period range remains unchanged until the last backoff, resulting in high packet collisions that causes a longer delay. The average packet delivery delay of PLA-MAC is increased after the fifth BMSN and gradually rises until the fourteenth BMSN. However, this degraded performance is not acceptable in a real-time emergency situation.

In an emergency situation, eMC-MAC also assigns very low backoff period range to the BMSNs with emergency packets. Instead, the BMSNs with low priority data packets get higher backoff period range during contention. Therefore, the traffic of the BMSNs with low priority data packets is delayed. The backoff period range is assigned to BMSNs with emergency data packets repetitively used in the backoff period ranges of all backoffs, and every backoff period range starts from zero. The results show that the BMSNs with low priority data packets can access the channel before the BMSNs with emergency data packets. Thus, emergency data packets delay. Moreover, the assigned backoff period ranges remain unchanged in all backoffs which also increases collision and packet delivery delay. In eMC-MAC, the average packet delivery delay increases gradually and becomes worse after 11^th^ BMSN. PG-MAC shows very high delay. This is because PG-MAC assigns [0–4] as a backoff period range to the BMSNs with emergency packets and this backoff period range is repetitively used in all the backoff period ranges. Again, all the backoff period ranges start from zero and remain unchanged in all backoffs. Therefore, the collision rate increases and consequently, the retransmission rate also increases resulting in longer delay. In normal situation, the TCP-CSMA/CA scheme of eTA-MAC protocol observes the lowest average packet delivery delay in normal situations because each traffic class gets a distinct, minimized, and prioritized backoff period range in every backoff. Even in the last backoff, the upper limit of the backoff period range for lowest traffic class is 31 which reduces packet delivery delay of BMSNs belonging to the lowest level TC. Thus, TCP-CSMA/CA scheme of eTA-MAC reduces the average packet delivery delay and attains improvement of 58%, 23%, 59% as compared to the PLA-MAC, eMC-MAC, and PG-MAC schemes, respectively.

In case of an emergency situation ETC is created dynamically. Moreover, a distinct and lowest backoff period range is assigned to BMSNs with emergency data packets, which belongs to ETC in every backoff. Hence, BMSNs with EDPs transmit data packets with lower delay. Also, BMSNs that belong to CTC, RTC, DTC, and NTC get distinct, minimized, and prioritized backoff period range in every backoff. Even the BMSNs that belong to the lowest priority traffic class, i.e., NTC get very low backoff period range, i.e., [32–35] even in its last backoff. Therefore, the BMSNs with lower priority data packets also observe the lower delay comparatively. Thus, ETCP-CSMA/CA scheme of the eTA-MAC reduces the packet delivery delay and attains improvement by 52%, 10%, 54% against PLA-MAC, eMC-MAC, and PG-MAC, respectively.

**Fig 4:**
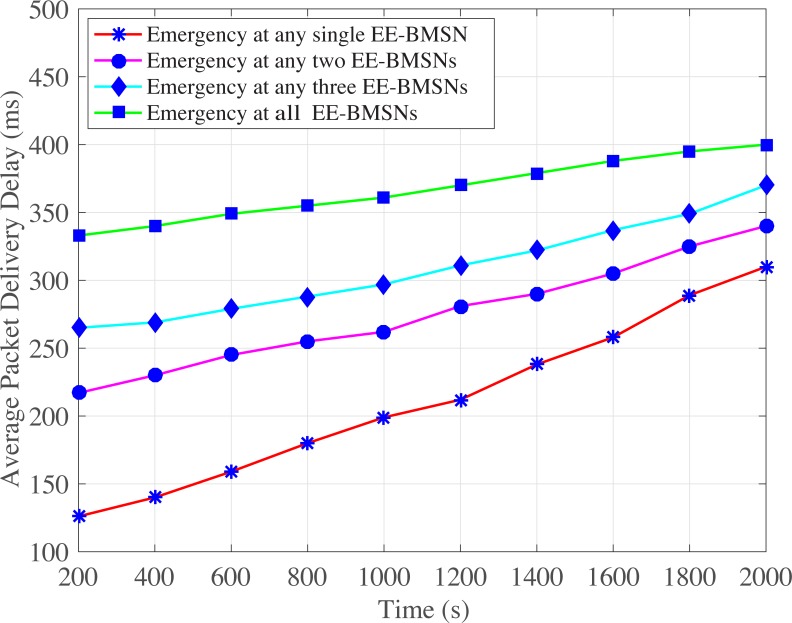
Average packet delivery delay versus time in seconds.

In addition, (Fig 4) shows the average packet delivery delay comparison among emergency occurrence at single or multiple EE-BMSNs. The emergency at any single EE-BMSN comparatively shows lowest average packet delivery delay. This is because the emergency data packets are generated by only one EE-BMSN, and ETCP-CSMA/CA assigns the distinct and lowest backoff period range to that particular EE-BMSN in each backoff. Hence, its average packet delivery delay is comparatively decreased due to less traffic load. Emergency at any two EE-BMSNs presents the performance of all possible combinations of simultaneous occurrences of emergency at any two EE-BMSNs. It shows a little bit higher average packet delivery delay due to emergency traffic generation at two EE-BMSNs. Even though ETCP-CSMA/CA assigns the lowest backoff period range to these two EE-BMSNs but the assigned backoff period range is shared by these two EE-BMSNs which results in collision. This collision increases the average packet delivery delay. Similarly, average packet delivery delay is increased by increasing the number of EE-BMSNs that generate emergency data packets. Therefore, Emergency at any three EE-BMSNs shows higher delay as compared to Emergency at any two EE-BMSNs. Emergency at all EE-BMSNs shows highest average packet delivery delay because all EE-BMSNs generate emergency data packets which increases the traffic generation rate. Consequently, network traffic load is increased which results in collision. The retransmission of collided data packets increases the packet delivery delay.

**Fig 5:**
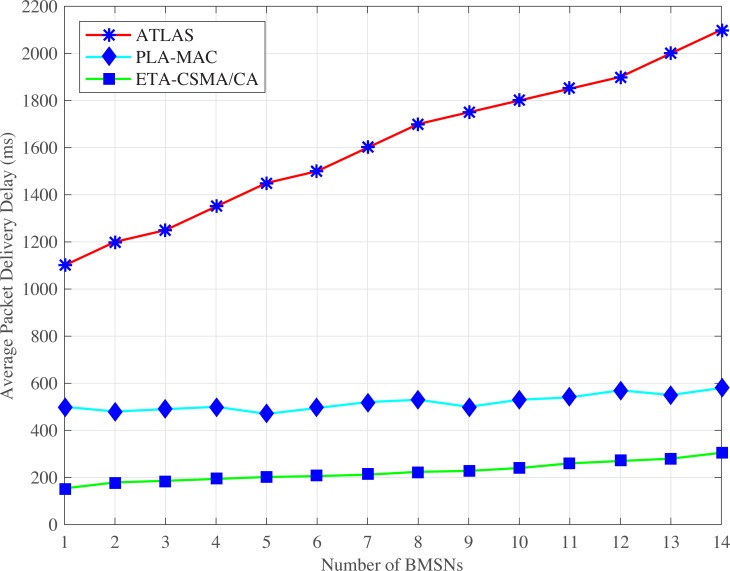
Average packet delivery delay versus number of BMSNs.

**Fig 6:**
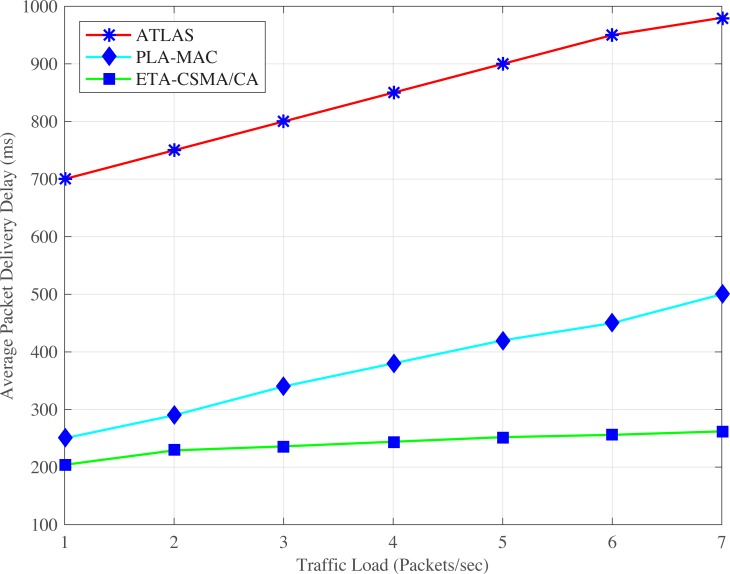
Average packet delivery delay versus varying number of packets/second.

ATLAS shows very high average packet delivery delay as shown in Figs 5 and 6. In ATLAS, traffic prioritization is ignored. Therefore, all BMSNs contend to access the channel using the common backoff period range in each backoff which results in a high collision. This high collision increases the retransmission rate resulting in longer packet delivery delay. In addition, as shown in (Fig 6), the increasing number of data packets per second results in a high collision rate, which gradually increases the average packet delivery delay. Therefore, the emergency and even normal data packets become dead before transmission due to this high packet delivery delay. ATLAS does not provide any lifetime management system to avoid the transmission of dead data packets. This puts an additional traffic load over the network. The extra traffic load causes more increases in the packet delivery delay. Therefore, as shown in (Fig 5), the average packet delivery delay of ATLAS increases continuously by increasing the number of BMSNs. Hence, the dynamic traffic adjustment of ATLAS does not improve the average packet delivery delay.

Similarly, PLA-MAC does not provide any lifetime management system for emergency and normal data packets in case of an emergency. This causes high collision rate increase the retransmission rate as well as the average packet delivery delay. As shown in (Fig 5), the overall average packet delivery delay of PLA-MAC is very high and becomes worse after ninth BMSN. It then gradually rises till the fourteenth BMSN. In (Fig 6), depicting PLA-MAC, as the traffic load rises by increasing the number of data packets per second at each BMSN, the collision rate increases. However, the traffic adaptivity provided by PLA-MAC does not improve the average packet delivery delay which is required in real-time WBANs. Our proposed eTA-MAC protocol observes very low average packet delivery delay because it stops the transmission of dead data packets over the network and thereby, decreases the unnecessary traffic load over the network. Alive data packets get the opportunity to transmit on time with lower delay. Hence, in (Fig 5), the ETA-CSMA/CA scheme of eTA-MAC improves the average packet delivery delay by 86% and 57% compared to ATLAS and PLA-MAC respectively. In addition, the ETA-CSMA/CA scheme of eTA-MAC achieves an average packet delivery delay which is 72% better than ATLAS and 36% better than PLA-MAC at varying traffic load, in terms of a number of packets per second as shown in (Fig 6).

#### 4.2.2 Throughput

**Fig 7:**
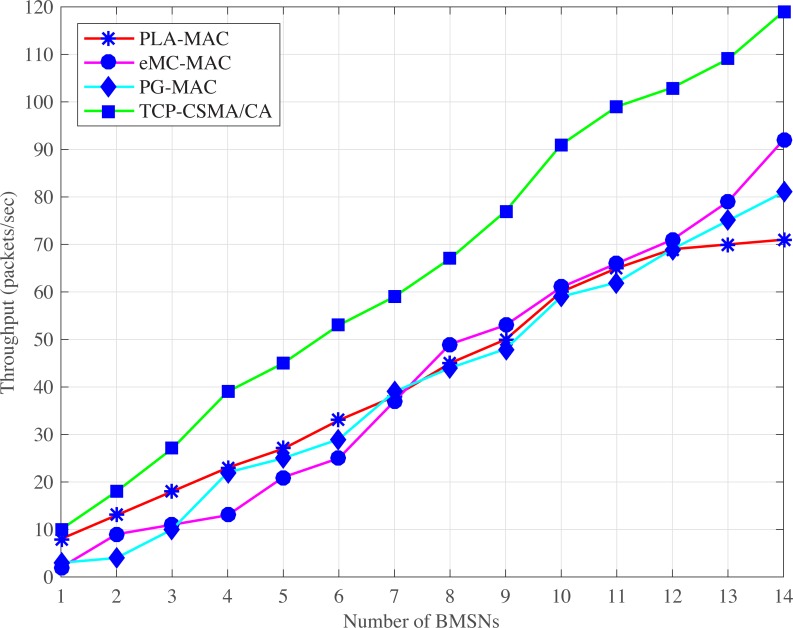
Throughput versus number of BMSNs in normal situation.

**Fig 8:**
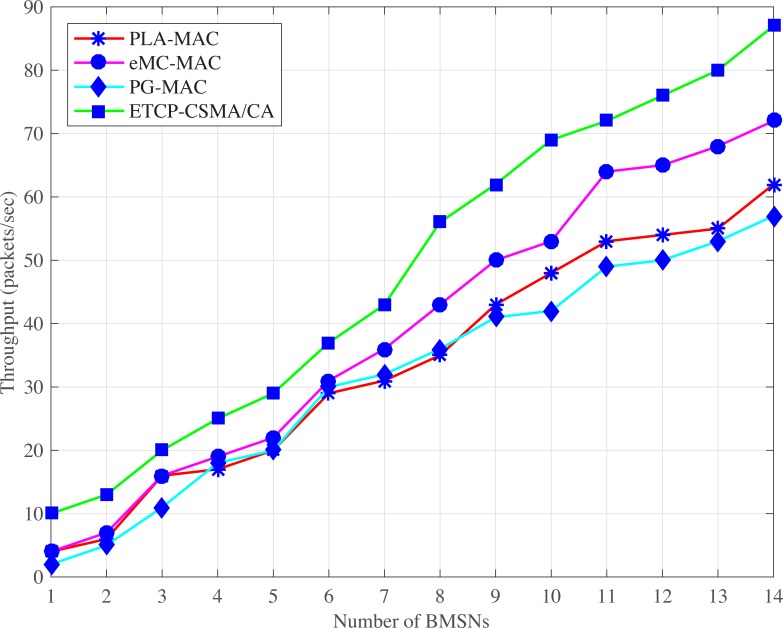
Throughput versus number of BMSNs in emergency situation.

Figs 7 and 8, illustrate PLA-MAC in which a distinct backoff period range is assigned to each traffic class in the first backoff whose range remains unchanged until the last backoff. However, the repetitive assignment of the same backoff period range in all backoffs increases the collision which results in more retransmission, and reduces the overall throughput of PLA-MAC. Similarly, in eMC-MAC, emergency packets use very low backoff period range which is used repetitively in all other backoff period ranges assigned to the BMSNs with different types of data packets. This repetitive use of a backoff period range increases the collision rate. In normal and emergency situations, eMC-MAC shows lower throughput up to the seventh BMSN because very low backoff period range is assigned to these BMSNs. The first seven BMSNs are with UPs, CPs or RPs. The BMSNs with CPs or RPs get zero as a backoff number during contention, and BMSNs with UPs or emergency data packets get [0–3] as a backoff period range. This results in high collision among these BMSNs.

In PG-MAC, very low backoff period ranges are assigned to BMSNs with ED, PD or ND in every backoff during contention. The traffic load increases due to the usage of very low backoff period ranges. The increased traffic load causes a high collision and decreases the throughput. Furthermore, the backoff period range assigned to BMSNs with EDs repetitively used in all the backoff period ranges that are assigned to BMSNs with PDs or NDs. Thus, the collision rate increases and thus, decreases the throughput of emergency data packets. The proposed eTA-MAC protocol performs better as compared to the PG-MAC because it creates ETC dynamically in an emergency situation. It also assigns a distinct, lowest and prioritized backoff period range to BMSNs with emergency or normal data packets in every backoff. In normal situations, the achieved throughputs of TCP-CSMA/CA scheme of eTA-MAC are 55% compared to PLA-MAC, 56% compared to eMC-MAC, and 61% as compared to PG-MAC. In emergency situations, the achieved performances of ETCP-CSMA/CA scheme of eTA-MAC are 43% as compared to PLA-MAC, 23% as compared to eMC-MAC, and 52% as compared to PG-MAC.

**Fig 9:**
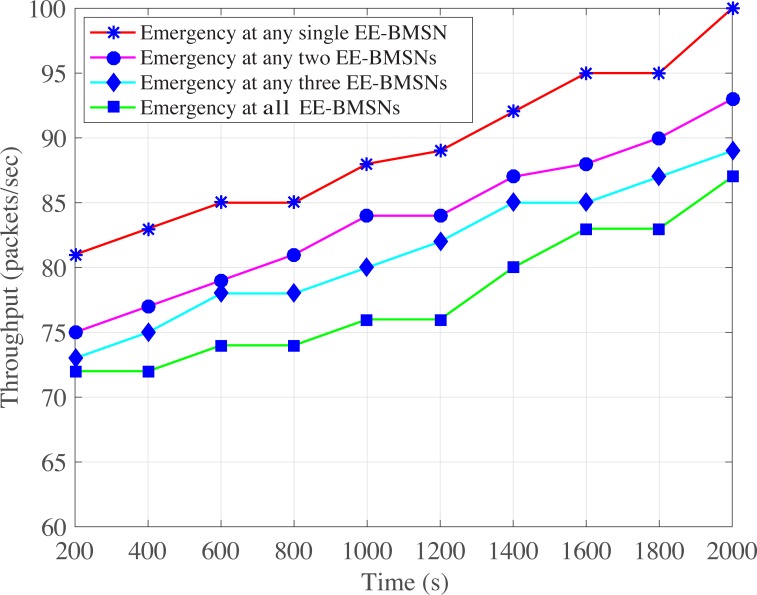
Throughput versus time in Seconds.

Additionally, (Fig 9) shows highest throughput in case of emergency occurrence at any single EE-BMSN. This is because only one EE-BMSN generates traffic at high rate due to emergency and ETCP-CSMA/CA comparatively assigns a distinct and lowest backoff period range to that particular EE-BMSN. The network throughput goes down in case of emergency at any two EE-BMSNs because now two EE-BMSNs generate traffic at high rate due to emergency and share the backoff period range assigned by ETCP-CSMA/CA. Similarly, the network throughput is decreased as more EE-BMSNs generate emergency traffic. Therefore, the emergency at any three EE-BMSNs presents lower throughput and Emergency at all EE-BMSNs shows the lowest throughput.

**Fig 10:**
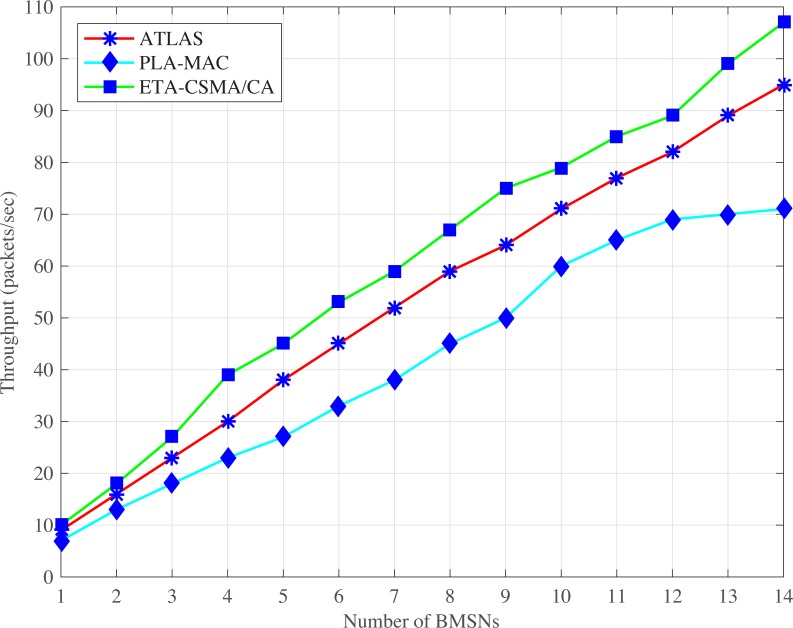
Throughput versus number of BMSNs.

**Fig 11:**
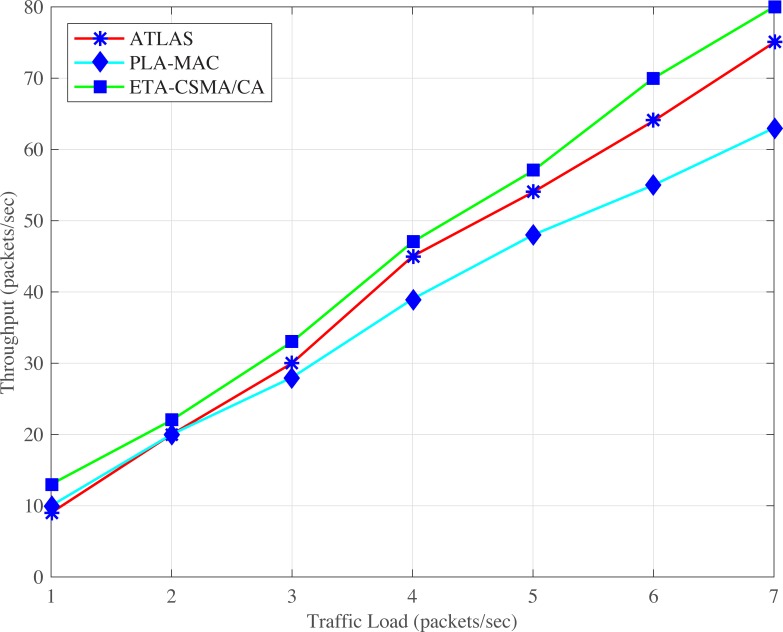
Throughput versus varying number of packets/second.

ATLAS does not provide traffic prioritization, and all the BMSNs, without distinction, use the same backoff period range in every backoff during contention. It creates unnecessary traffic load over the network which results in a high collision. The lifetime of the data packets expires before transmission due to this high collision. But ATLAS does not provide any mechanism to stop the transmission of dead data packets over the network which also creates unnecessary traffic load on the network, causing more increases in collision rate. Hence, the high collision rate also increases the retransmission rate and thereby, reduces the overall throughput of ATLAS, as shown in Figs 10 and 11. Thus, the dynamic traffic adjustment of ATLAS does not improve the overall throughput of the network.

Similarly, PLA-MAC does not manage the transmission of emergency and normal dead data packets in an emergency situation. Therefore, the overall throughput of the network is reduced in PLA-MAC and becomes worse after the eleventh BMSN as shown in (Fig 10). The throughput of PLA-MAC is better than ATLAS at 1 and 2 packets/second because it provides traffic prioritization as shown in (Fig 11). But due to the lack of a lifetime management system for emergency and normal data packets, and the assignment of specific backoff period range to each traffic class which remains unchanged in all backoffs, the packet collision rate is increased. The high collision rate also decreases the throughput of PLA-MAC at a variable number of packets/second. Therefore, the traffic adaptive approach of PLA-MAC does not improve the overall throughput of the network. The ETA-CSMA/CA of eTA-MAC protocol performs better in terms of throughput because it drops dead emergency and normal data packets in an emergency situation, which controls the traffic load over the network. In (Fig 10), the achieved throughputs of ETA-CSMA/CA of eTA-MAC protocol are 14% as compared to ATLAS and 45% as compared to PLA-MAC. Additionally, the ETA-CSMA/CA scheme of eTA-MAC achieves 9% and 23% higher throughput as compared to ATLAS and PLA-MAC, respectively, at varying traffic load, in terms of a number of packets per second as shown in (Fig 11).

#### 4.2.3 Energy consumption

**Fig 12:**
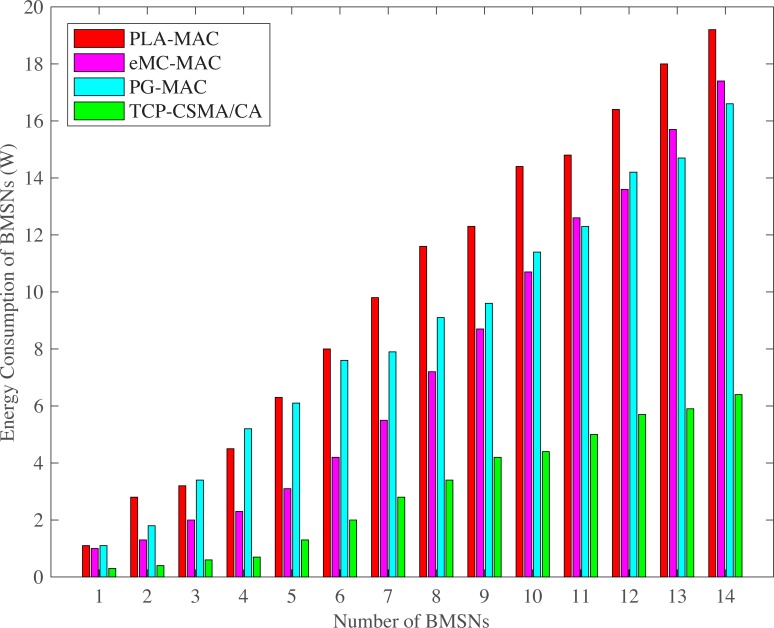
BMSNs energy consumption versus number of BMSNs in normal situation.

**Fig 13:**
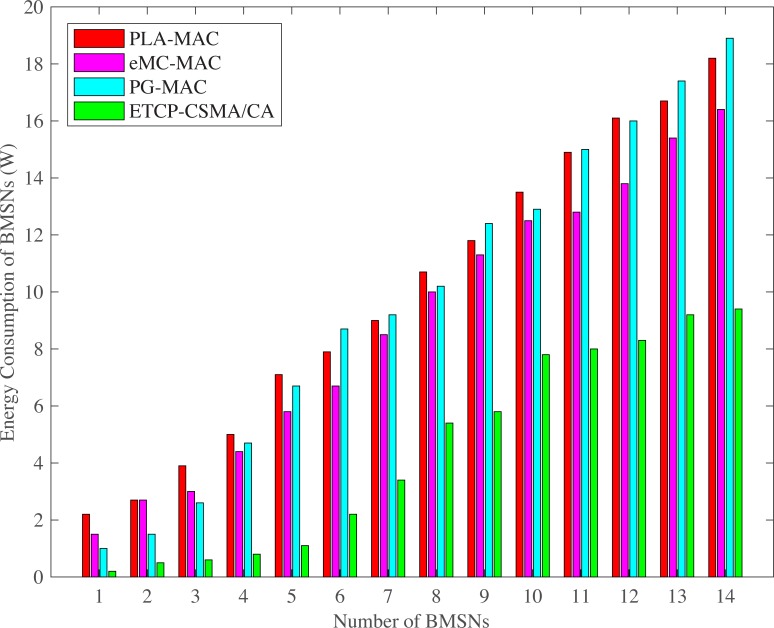
BMSNs energy consumption versus number of BMSNs in emergency situation.

The highest energy consumption of BMSNs is observed in PLA-MAC during normal and emergency situations as shown in Figs 12 and 13. However, in PLA-MAC, low priority traffic waits for an extended period to access the channel in the CAP, and the specific backoff period range is used repetitively by each traffic class in every backoff. It results in high collision among all kinds of BMSNs and consequently increases packet retransmission rate. Therefore, in PLA-MAC, the energy consumption of each BMSN is increased. PG-MAC also shows worse energy consumption than PLA-MAC in few of the BMSNs. This is because the backoff period ranges assigned to the BMSNs of different traffic types during contention are very low. It creates a very high collision, and the dropped data packets require retransmission, increasing the energy consumption of each BMSN. The eMC-MAC shows lower energy consumption as compared to PLA-MAC and PG-MAC, but in normal situations, it appears higher than PG-MAC at BMSNs 13 and 14. The reason is that in eMC-MAC very high backoff period ranges are assigned to BMSNs 13 and 14. The proposed eTA-MAC protocol reduces the energy consumption of BMSNs because it removes repetition in each backoff during contention, assigns prioritized and minimized backoff period range to each traffic class and allocates sufficient timing to each BMNS. This situation enables BMNS to contend and transmit data during normal or emergency situations. It also dynamically creates an emergency traffic class in emergency situations and removes emergency traffic class in case of emergency resolved. Thus, all BMSNs comparatively consume less energy. In normal situations, the TCP-CSMA/CA scheme of eTA-MAC protocol consumes 70% less energy as compared to PLA-MAC, 59% less than eMC-MAC and 64% less energy compared to PG-MAC. In an emergency situation, the ETCP-CSMA/CA scheme of eTA-MAC consumes 55% less energy as compared to PLA-MAC, 50% less then eMC-MAC and 54% less as compared to PG-MAC.

**Fig 14:**
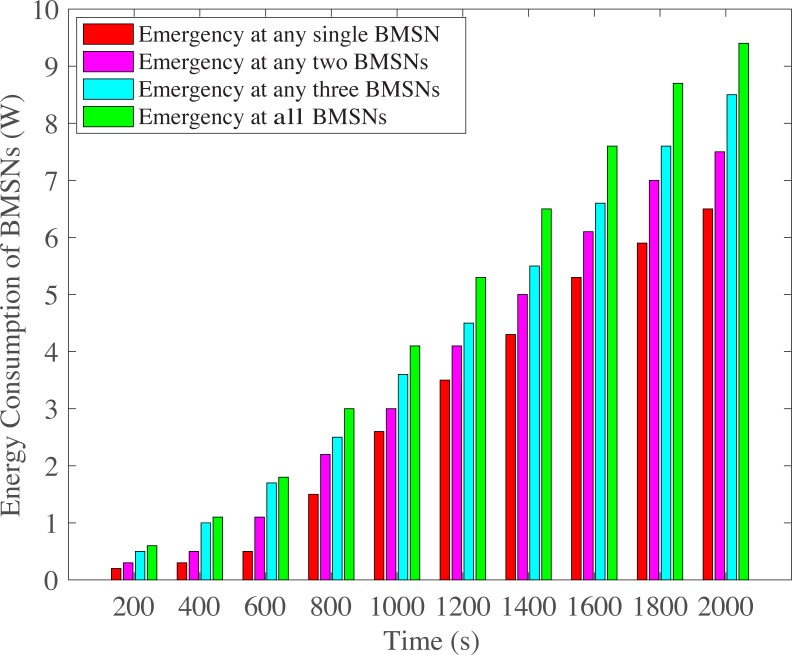
BMSNs energy consumption versus time in Seconds.

Fig 14 presents lowest energy consumption when emergency occurs at any single EE-BMSN because that single EE-BMSN faces no contention in its first backoff due to distinct and lowest backoff period range (that is assigned by ETCP-CSMA/CA). As number of EE-BMSNs are increased that generate emergency data packets, contention is increased which increases retransmission rate. As a consequence, the energy consumption is also increased. Therefore, Emergency at any two EE-BMSNs consumes more energy, Emergency at any three EE-BMSNs consumes even more energy and Emergency at all EE-BMSNs comparatively consumes highest energy.

**Fig 15:**
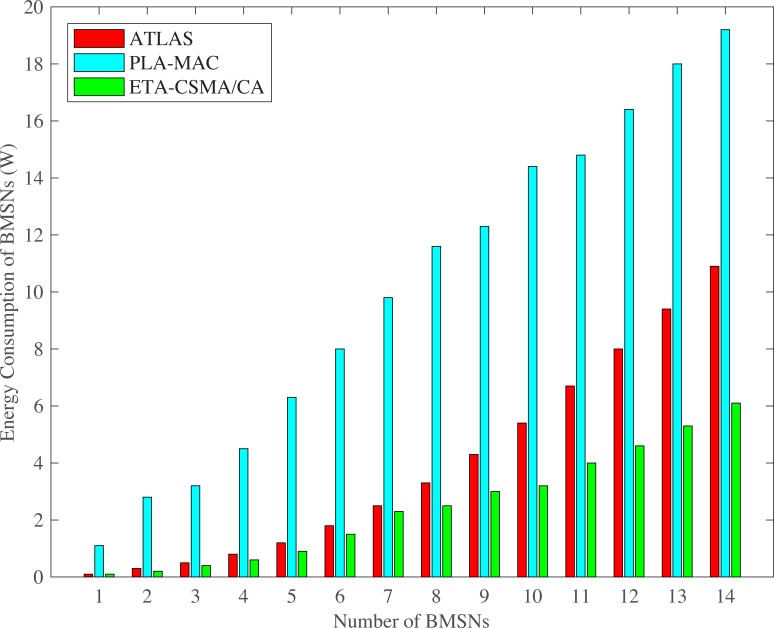
Energy consumption versus number of BMSNs.

**Fig 16:**
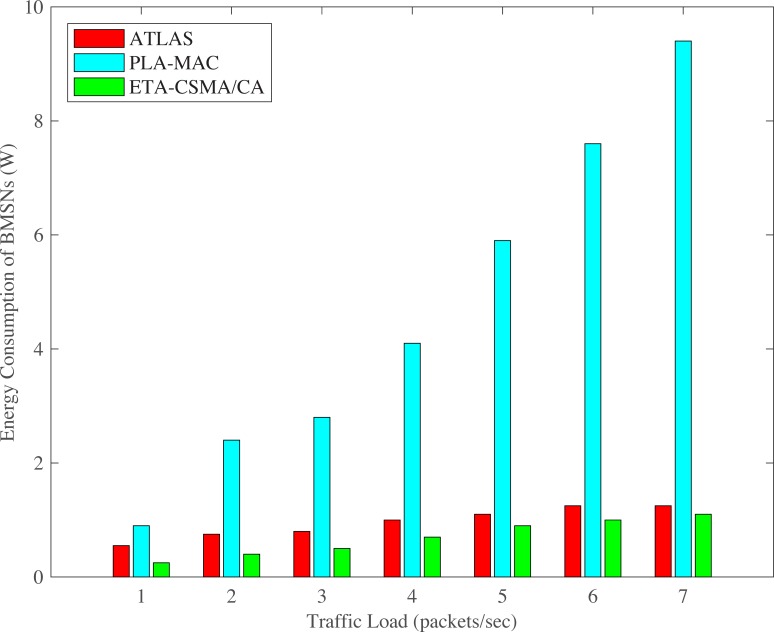
Energy consumption versus varying number of packets/second.

The ATLAS shows lower energy consumption of BMSNs as compared to PLA-MAC as shown in Figs 15 and 16. ATLAS shows higher energy consumption on varing number of BMSNs and transmission rates as compared to ETA-CSMA/CA scheme of eTA-MAC. This is because it does not provide traffic prioritization. All emergency and normal BMSNs use the common backoff period range in all backoffs during contention, which increases collision and retransmission rate. Due to this high collision, data packets become dead before transmission and this puts an unnecessary extra traffic load on the network. Furthermore, ATLAS does not have any mechanism to stop transmission of dead emergency and normal data packets in an emergency situation. All these deficiencies of ATLAS result in high collision and then high retransmission, thereby, increasing the energy consumption of BMSNs. Hence, ATLAS does not create a balance between energy consumption and throughput. It does not also minimize the energy consumption of BMSNs with normal data packets.

Additionally, PLA-MAC does provide a mechanism to stop the transmission of dead emergency and normal data packets in an emergency situation. Thus, as the number of packets per second increases, the collision rate also increases. In PLA-MAC, the data packet loss rate is increased due to the collisions. The lost data packets require retransmission which increases the energy consumption of each BMSN. Hence, PLA-MAC shows very high energy consumption. However, ETA-CSMA/CA scheme comparatively consumes less energy at each transmission rate because it stops the transmission of dead emergency and normal data packets in an emergency situation. Hence, upon varying the number of BMSNs, ETA-CSMA/CA consumes 37% less energy as compared to ATLAS and 76% less energy compared to the PLA-MAC. On varying the number of packets per second, ETA-CSMA/CA consumes 28% less energy as compared to ATLAS and 85% less energy compared to the PLA-MAC.

#### 4.2.4 Accuracy of Emergency Monitoring

**Fig 17:**
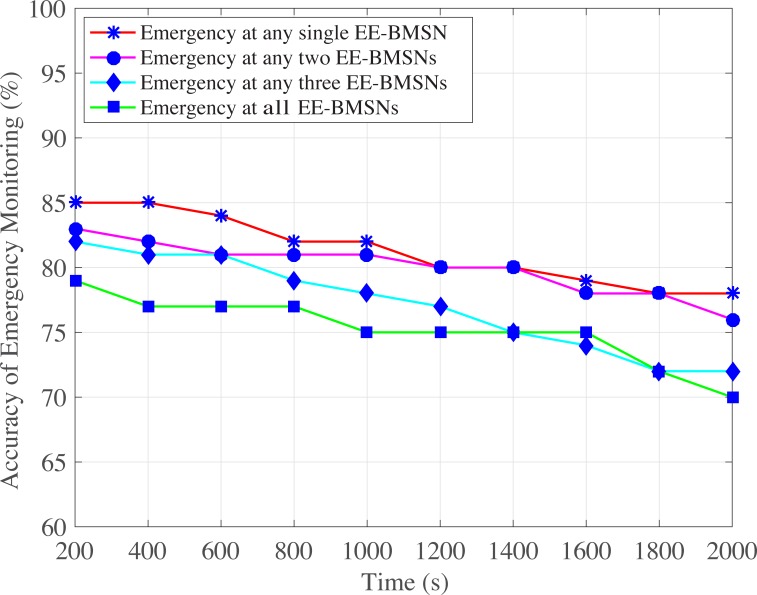
Accuracy of emergency monitoring versus time in seconds.

Fig 17 depicts the accuracy of emergency monitoring at single or multiple EE-BMSNs. The highest accuracy of emergency monitoring is observed in case of emergency at single BMSN comparatively which goes down gradually by increasing the simulation time. In fact, network traffic load is increased when emergency occurs at single EE-BMSN which is increased more by increasing simulation time. That high network traffic load raises packet collision rate which reduces the accuracy of emergency monitoring. Similarly, network traffic increases more in case of emergency at any two EE-BMSNs. However, traffic generation rate is increased in case of emergency. In addition, that higher traffic generation rate becomes more worse in case of long simulation time. Thus, accuracy is reduced when more EE-BMSNs generate emergency data packets and by increasing simulation time.

## 5 Conclusion

The purpose of the current study was to provide contention-based prioritized channel access to heterogenous-natured BMSNs in normal or sporadic emergency situations with emergency-based traffic adaptive approach. All types of BMSNs either with emergency or normal traffic must perform contention to access the channel in CAP. There is a problem in the design of IEEE 802.15.4 based slotted-CSMA/CA which raises three issues during contention that need to be resolved. First, the heterogeneous-natured patient’s vital-signs traffic need prioritized channel access. Second, is sporadic emergency traffic which requires instantaneous transmission with minimum delay and packet loss without ignoring the normal traffic. Third, there is the need for a dynamic adjustment of traffic in order to accommodate the variations in heterogeneous traffic rates due to an emergency situation with a balance between throughput and energy. Moreover, the BMSNs with normal traffic require energy preservation. The developed eTA-MAC protocol offers a more realistic solution in line with the patient’s body and proves to be better than the benchmark protocols in terms of packet delivery delay, throughput, and energy consumption.

## Supporting information

S1 File This data set is used for Figs 2, 7 and 12. (XLSX)

S2 File This data set is used for Figs 5, 10 and 15. (XLSX)

S3 File This data set is used for Figs 4, 9,14 and 17. (XLSX)

S4 File This data set is used for Figs 6, 11 and 16. (XLSX)

S5 File This data set is used for Figs 3, 8 and 13. (XLSX)
